# The regulation roles of *miR-125b*, *miR-221* and *miR-27b* in porcine Salmonella infection signalling pathway

**DOI:** 10.1042/BSR20160243

**Published:** 2016-08-31

**Authors:** Min Yao, Weihua Gao, Jun Yang, Xiongyan Liang, Jingbo Luo, Tinghua Huang

**Affiliations:** * College of Animal Science, Yangtze University, Jingzhou, Hubei 434025, China

**Keywords:** *miR-125b*, *miR-221*, *miR-27b*, pig, Salmonella infection

## Abstract

miRNAs are non-coding RNA molecules typically 18–22 nucleotides long that can suppress the expression of their target genes. Several laboratories have attempted to identify miRNAs from the pig that are involved in Salmonella infection. These bioinformatics strategies using the newly available genomic sequence are generally successful. Here, we report an *in silico* identification of miRNAs in pig focusing on the Salmonella infection pathway, and further investigated the differential expression of those miRNAs by quantitative real-time PCR during pre- and post-natal stage of Salmonella inoculation from the peripheral blood of commercially breed pigs. We identified 29 miRNAs that have predicted targets in the Salmonella infection pathway and nine of them were not yet described in pig. In addition, the expression of nine selected miRNAs was validated in the peripheral blood by northern blotting. Through expression analyses, differences were found between pre- and post-natal stages of Salmonella inoculation for *miR-221*, *miR-125b* and *miR-27b*—all of them were suppressed 2 days after Salmonella inoculation. The predicted targets of those three miRNAs were validated by luciferase reporter assays. We show that FOS is a direct target of *miR-221*, *miR-125b* can suppress MAPK14, and *miR-27b* can target IFNG. These findings will be helpful in understanding the function and processing of these miRNAs in Salmonella infection. The miRNA differentially expressed in the peripheral blood of commercial breed pigs suggest that it can be used as genetic markers for salmonella infection resistance in pigs.

## INTRODUCTION

Salmonella colonization of pigs can lead to enterocolitis, and the bacteria often establish a carrier status in the host [1]. Salmonella colonization is subclinical but can decrease the quality of pork, and thus has a negative economic impaction on the meatpacking industry. Moreover, hogs that persistently shed Salmonella pose a significant threat to public health by increasing the potential for foodborne disease [1–3]. Reducing the incidence and severity of salmonellosis requires identification of the immune genes and pathways responsible for enhanced disease resistance and pathogen clearance [4]. In addition to protein-coding genes, miRNAs play important roles in the regulation of Salmonella infection. The miRNAs are one of the most important families of noncoding RNAs (ncRNAs), and they have important sequence-specific post transcriptional regulators of gene expression [5]. miRNAs are expressed in a wide range of eukaryotic organisms and suppress the expression of target gene by binding to the 3’ UTR of their target genes [5,6]. The mammalian inflammatory response to Salmonella infection involves the induction of several miRNAs. This process must be carefully regulated to achieve pathogen clearance and prevent the consequences of regulated expression.

Previous studies found that *miR-146* and *miR-155* were co-induced in macrophage cells in response to microbial LPS [7–9]. In human monocytes, the *miR-146* and *miR-155* can respond to endotoxins and a variety of microbial components and pro-inflammatory cytokines [9]. Analysis of host miRNA by high throughput sequencing confirmed the co-induction of *miR-146* and *miR-155* upon live microbial infection with wild type (WT) *Salmonella Typhimurium* strains [10]. The *miR-146* family members are known as inflammation inducible miRNAs involved in the negative feedback regulation of toll-like receptor (TLR) signalling [9]. In zebrafish, *miR-146a* and *miR-146b* were commonly induced by infection of embryos with Salmonella Typhimurium [11]. Knockdown of TRAF6 and mutation of MYD88 showed that the induction of *miR-146a* and *miR-146b* by Salmonella Typhimurium infection was affected by disruption of the MYD88-TRAF6 pathway, which mediates the transduction of the TLR signals and cytokine responses [11]. The *miR-155* can target the TLR/IL-1 inflammatory pathway and serves as a negative feedback loop to modulate inflammatory cytokine production in response to microbial stimulation [12]. Recent studies have showed that *miR-155* is differentially expressed in pig during Salmonella Typhimurium infection [13].

In addition to *miR-146* and *miR-155*, the let-7, *miR-15*, *miR-128* and *miR-29a* also have roles in Salmonella infection. Schulte et al. [14] demonstrated that the let-7 family of miRNAs was down-regulated in macrophages infected with Salmonella Typhimurium or challenged with purified LPS. let-7 down-regulation relieved suppression of the major macrophage cytokines IL-6 and IL-10 [14,15]. Maudet et al. demonstrated that several members of the *miR-15* miRNA family were down-regulated during Salmonella infection due to the inhibition of the transcription factor E2F1 [16]. Analysis of the *miR-15* family targets revealed that de-repression of CCND1 and the consequent promotion of G1/S transition are crucial for Salmonella intracellular proliferation. Zhang et al. [17] showed that Salmonella can up-regulate intestinal epithelial *miR-128* expression, which in turn decreases levels of epithelial cell-secreted CSF1. This was correlated with the level of Salmonella infection. Hoeke et al. [18] showed that *miR-29a* was up-regulated after Salmonella infection and was a main regulator of the focal adhesion pathway and the actin cytoskeleton pathway.

However, these miRNAs do not fully explain Salmonella infection in pig. In the present study, we report a computational prediction to identify the miRNAs that involved in porcine Salmonella infection pathway. The expression levels of these miRNAs in peripheral blood during the pre- and post-natal stages of Salmonella infection were investigated by quantitative real-time PCR. The targets of differentially expressed miRNAs were further validated using a luciferase reporter assay. The miRNAs identified here and their targets in pigs are valuable for understanding the molecular mechanism of miRNAs in Salmonella infection.

## MATERIALS AND METHODS

### Prediction and analysis of miRNA binding site

The porcine mRNA 3’ UTR sequences were collected from the Ensemble database using the Biomart toolkit (http://www.biomart.org/). There are 68 genes in the Salmonella infection pathway, and 44 have annotated unique UTRs. The porcine 3’ UTRs were added to the 23 genome vertebrate sequence alignments downloaded from the TargetScan website (http://www.targetscan.org) using the multiple sequence alignment tool MUSCLE [19,20] (http://www.drive5.com/muscle/). Finally, we built a 24 way 3’ UTR alignment including the porcine 3’ UTR sequence (Supplementary Document 1). The miRNA data set comprised all 2659 mature miRNA sequences of the miRBase entries (human, mouse and pig, release 21). These mature sequences were grouped into 1781 families with both porcine miRNA and other vertebrates (human and mouse) miRNA sharing of the same seed sequence (Supplementary Document 2). TargetScan [21,22] and miRBase [23–25] identified potential binding sites for miRNAs in protein-coding genes involved in the Salmonella signalling pathway.

### Homology searches and secondary structure predictions

We used the miRNA precursor (pre-miRNA) sequence obtained from human, mouse and pig for Blast searches against the swine genomic sequence. The BLASTN algorithm (optimized for ‘somewhat’ similar sequences) was used to find homologous miRNA sequences for pig that matched for possible sequence variations [26,27]. Genomic sequences that matched pre-miRNAs were selected, and the flanking 20 nucleotides were excised. Sequence folding was performed using the program Mfold version 3.5 [28,29]. Pre-miRNA sequences were considered potential miRNA when fitting the following criteria: (1) the predicted mature miRNA had no more than four nucleotide substitutions when aligned with known human and mouse mature miRNAs; (2) the RNA sequence folded into an appropriate stem-loop hairpin secondary structure and (3) a mature miRNA sequence site was present in one arm of the hairpin structure. In addition, we submitted the new pre-miRNA sequences to a SVM-based program called miRFinder [30] that identifies pre-miRNA hairpin sequences and classifies them as real or pseudo pre-miRNAs.

### Northern blot analysis of miRNA primary transcript and mature sequence

For chemiluminescent photography assays, the reverse transcription products were separated by 15% polyacrylamide gel electrophoresis and then transferred to Hybond N+ membranes (Amersham) using a semidry blotter. The membranes were UV-cross-linked and baked for 30 min at 80°C. The DIG-labelled DNA oligonucleotides complementary to small RNA sequences were used as the probes. The membranes were hybridized overnight with the probes at 42°C within the hybridization buffer. The membranes were then washed with wash buffer three times at 42°C. The DIG-labelled probes were immunodetected with anti-digoxigenin-AP and then incubated with the chemiluminescence substrate CSPD. The membranes were exposed to X-ray films for 15–25 min.

### Quantitative real-time PCR of mature miRNA

The quantitative real-time PCR approach was used to measure the expression level of miRNAs in peripheral blood samples during Salmonella inoculation in pig as described below. Ten peripheral blood samples were divided into pre- and post-natal (2 h) stages of Salmonella inoculation were collected (commercial breed). The ethics committee of Yangtze University approved the study. Total RNA from swine peripheral blood samples was isolated using the PAXgene blood miRNA kit (Qiagen, 763134) according to the manufacturer's protocol. The RNA concentration was determined with a NanoDrop 10000 spectrophotometer, and the RNA integrity was evaluated using the Agilent 2100 Bioanalyzer. RNA integrity number (RIN) was determined using the RIN algorithm of the Agilent 2100 expert software. Samples with an RIN ≥7 were included in the present study.

The quantitative RT-PCR was performed on a thermal cycler ABI 7500 with real-time PCR (Applied Biosystems) using the pincer probes [31]. The amplification conditions for all reactions were 95°C for 2 min, 40 cycles of denaturation at 95°C for 15 s, and extension at 58°C for 15 s. The primers used were listed in Supplementary Document 3. The experimental design was randomized with five replicates per time point. Importantly, all reverse transcriptase reactions were run with ‘no-template controls’. The no-template controls gave non-detectable signals in all samples and confirmed the high specificity of the miRNA quantification assay. The data were initially normalized using the 5S rRNA (121 nt) and U6 snRNA (45 nt). However, normalization with either of those two RNA levels or the average of these RNA levels did not correct any systematic bias between samples (as measured by reduced variance). Thus, results were normalized only to the total input RNA or cDNA used similar to our prior report as [13]. For each target gene, the comparison of expression values (Ct) of treatment levels (between day 0 and day 2) was performed using Student's *t* test. All statistical procedures were performed using R 3.0 for Windows.

### Luciferase reporter assay

For target genes, a 200–500 nucleotide 3' UTR segment including the miRNA target sites was inserted downstream of a Renilla luciferase ORF, and luciferase activity was compared with that of an analogous reporter with point substitutions disrupting the target sites. Two mutation sites for each miRNA binding site were tested. The logic behind the luciferase reporter assay is that mutation of a miRNA binding site should allow the up-regulation of target genes; hence, the target gene should be expressed differently between the WT and mutated constructs. For the luciferase reporter assays, we used the same protocol as described previously [32]. The comparison of luciferase values of each construct (vectors) (between WT and mutant, control and mutant) was performed using Student's *t* test.

## RESULTS

### MiRNAs predict target genes involved in the Salmonella infection pathway

**Figure 1:**
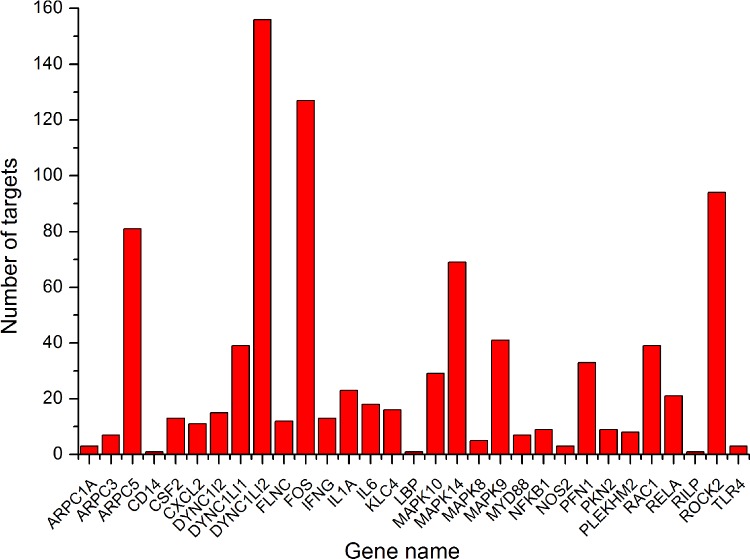
Numbers of predicted target sites per gene in the Salmonella infection signalling pathway

Porcine miRNA targets that were conserved across multiple organisms were identified with TargetScan searches for segments of 2–8 bases from the 5’ end of the miRNA using 3’ UTR alignments, which is referred to as the ‘miRNA seed’ (use default parameter). Complementarity matches were referred to as ‘seed matches’. TargetScan identified 907 putative miRNA sequences with target interactions that were conserved above the background levels. The 907 miRNA seed matches the 3’ UTRs of just 31 distinct genes because many genes were hit by multiple miRNAs. Those genes are under selective pressure to maintain multiple binding site pairings to multiple miRNAs. The miRNAs from the same miRNA family often have the same seed sequence. However, in cases where the same 3’ UTR was hit by multiple miRNAs from different families, the target sites generally did not overlap. This indicates that simultaneous binding and regulation of some of those genes use a combination of miRNAs. A complete list of the 907 target genes and the corresponding miRNAs is provided (Supplementary Document 4). The number of predicted target sites per gene is shown in Figure 1.

### Identification of the genomic sequence and prediction of secondary structure

We used all the mature miRNA sequences as the query and searched the binding site in the 3’ UTR of genes involved in the Salmonella infection pathway. In miRBase, the porcine miRNA dataset comprised 411 mature miRNA sequences entries, and some of the conserved miRNAs remain unknown (release 21). A computational identification of these conserved miRNA sequences was performed using the newly acquired porcine genomic sequence. We found 189 matches in the porcine genome using the 716 precursor sequences of miRNAs which predicted target genes in the Salmonella infection pathway from human and mouse as the query (Supplementary Document 5). A literature survey was performed to identify the miRNAs expressed in tissues related to Salmonella infection such as lymph node and blood tissues. A total of 19 miRNA sequences were expressed in lymph node, and 18 miRNA were expressed in peripheral blood (Table 1). Based on these miRNA candidates, their sequences were classified as real precursors by miRFinder [30].

**Table 1:** Predicted miRNAs involved in salmonella infection pathway and expressed in lymph node and blood

Name	Pig accession	Chromosome	Lymph node (human, Ct)	Blood (pig, copy number)
let-7a	ssc-let-7a	3	–	1001.88
let-7f	ssc-let-7f	3	–	305.99
let-7c	ssc-let-7c	13	–	242.43
*miR-103*	ssc-*miR-103*	16	–	521.67
*miR-125b*	ssc-*miR-125b*	9	25.46	637.06
*miR-128*	ssc-*miR-128*	15	–	3737.75
*miR-133b*	-	7	29.93	–
*miR-142-3p*	-	12	22.09	–
*miR-142-5p*	ssc-*miR-142-5p*	12	31.38	34928.13
*miR-145*	-	2	28.44	–
*miR-16*	ssc-*miR-16*	–	–	2050.11
*miR-15b*	ssc-*miR-15b*	13	27.70	1813.47
*miR-106a*	-	X	29.67	–
*miR-181b*	-	10	28.43	–
*miR-181a*	-	1	29.40	–
*miR-181c*	ssc-*miR-181c*	2	34.47	293.87
*miR-19b*	ssc-*miR-19b*	11	25.05	2616.53
*miR-19b*	ssc-*miR-19b*	X	–	2616.53
*miR-222*	-	X	27.24	–
*miR-221*	-	X	30.12	–
*miR-26a*	ssc-*miR-26a*	13	–	3304.35
*miR-27b*	ssc-*miR-27b*	10	28.59	–
*miR-28-3p*	ssc-*miR-28-3p*	–	–	1136.19
*miR-30c*	ssc-*miR-30c*	–	26.03	–
*miR-30e*	–	6	28.71	–
*miR-30b*	ssc-*miR-30b-5p*	–	26.89	2019.91
*miR-30a*	ssc-*miR-30a-5p*	–	–	1070.83
*miR-425*	ssc-*miR-425-5p*	13	34.96	4033.04
*miR-99a*	ssc-*miR-99a*	13	27.25	1367.07

### Analysis of miRNA pre and mature sequence using northern blot

**Figure 2:**
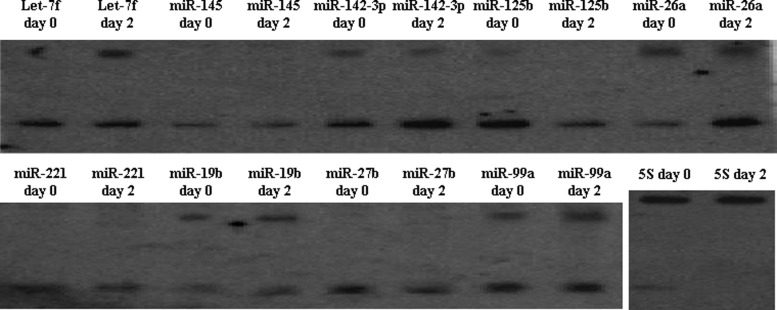
Northern blot analysis of pre and mature miRNA sequence Total RNA was isolated from peripheral blood sample collected at day 0 and day 2 of Salmonella inoculation. The 5S rRNA served as a loading control. Hybridization signals were detected for both pre-miRNAs and mature miRNAs.

*In vivo* expression analysis provides additional evidence for the existence of the miRNA candidates. To experimentally confirm and validate the results obtained from the *in silico* analysis, we examined the expression of nine miRNAs. These miRNAs were selected based on their reported biological functions, and their expression was verified by northern blotting hybridization in pig peripheral blood RNA samples, i.e. the normal pig peripheral blood RNA samples and peripheral blood RNA samples collected at 2 days after Salmonella inoculation. The hybridization of *miR-145*, *miR-125b*, *miR-221* and *miR-27b* showed signals that were approximately 22 nt in size, whereas the let-7f, *miR-142*, *miR-26a*, *miR-19b* and *miR-99a* detected not only the mature form but also the approximately 75-nt precursors on the same blots (Figure 2).

### Detection of differentially expressed miRNAs using quantitative real-time PCR

**Figure 3:**
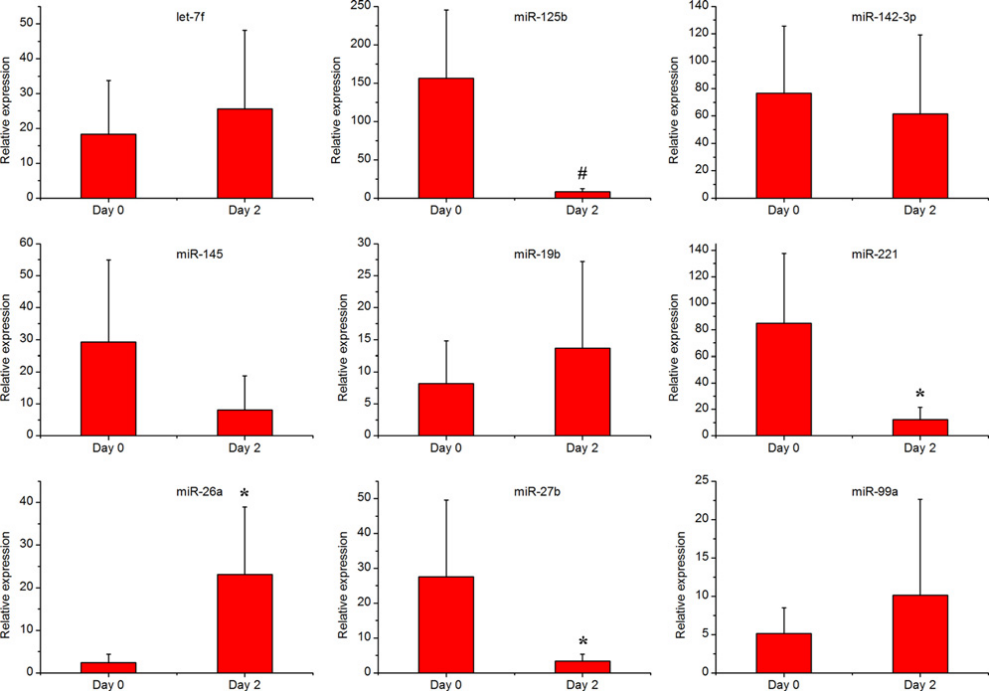
Expression of the nine selected miRNAs in response to Salmonella inoculation Day 0 and day 2 are time points before Salmonella inoculation and 2 days post Salmonella inoculation. The bar graphs show the relative expression level of the miRNAs detected by the pincer probe assay (*n*=5). Statistically significant differences compared with the day 0 group are visualized as *P≤0.05; ^#^*P*≤0.01.

Blood RNA samples from five animals inoculated with Salmonella bacteria were analysed via real-time PCR. The animal inoculation experiment used the method described previously in our research [13]. The expression levels of nine miRNAs were quantified using the pincer probe microRNA assay. All raw data and statistical results of these real-time PCR tests are available in Supplementary Document 6. Figure 3 shows that Salmonella inoculation down-regulated *miR-125b*, *miR-221*, *miR-27b* and up-regulated *miR-26a*. The expression of *miR-125b* was down-regulated 18-fold at 2 days of Salmonella inoculation (*P*<0.01). The expression of *miR-221* was down-regulated 6.9-fold at 2 days of Salmonella inoculation (*P*<0.05). The expression of *miR-27b* was down-regulated 8-fold in at 2 days after Salmonella infection (*P*<0.05). The expression of *miR-26a* was up-regulated 14-fold in at 2 days after Salmonella infection (*P*<0.05). Expression levels of the remaining miRNAs were not significantly different.

Uthe et al. have studied the bacteria in swine faecal samples in an independent population. All pigs were shedders of Salmonella bacterial at 2 dpi with an increase in body temperature (fever) [33]. We are interested in finding early responses that are correlated with Salmonella infection. We chose this particular stage (2 dpi) of the infection because our previous research has repeatedly shown that the peak of clinical symptoms (fever, diarrhoea, decreased appetite) as well as Salmonella shedding occurs at 2 days of post-inoculation.

Because changes in specific cell populations could account for the expression level response of miRNAs rather than changes in cell specific steady state RNA levels, we tested correlation of cell type and numbers (a standard complete blood count data including lymphocyte, monocyte, neutrophil, eosinophil and basophil counts) with the miRNA expression differences. No miRNAs had expression that was correlated with cell type numbers indicating that the miRNA expression differences detected by the real-time PCR cannot be explained by changes in numbers of major cell types.

### Experimental support for predicted regulatory targets

**Figure 4:**
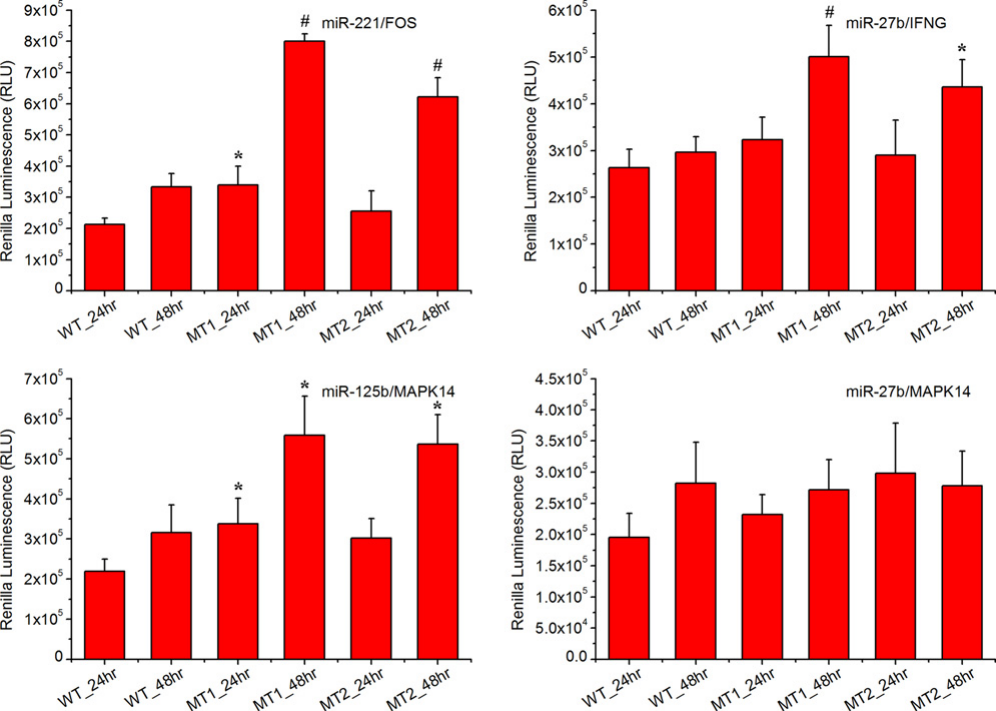
Experimental validation of the predicted targets in macrophage cells The WT construct had the 3’ UTR fragment of the target gene containing the predicted miRNA binding sites inserted within the Renilla luciferase 3’ UTR. The mutant (MT1 and MT2) construct was identical with the WT construct except that it had a point substitution disrupting binding site pairing to each miRNA seed. The bar graphs show the luciferase activity after the reporter plasmids were transfected into macrophage cells. Statistically significant differences compared with WT construct are visualized as *P≤0.05, ^#^*P*≤0.01.

Reporter assays were used to test three predicted targets (FOS, IFNG and MAPK14) of mammalian miRNAs in macrophage cells (RAW 264.7). The reporter vector containing the WT and mutated 3’ UTR and expressing Renilla luciferase were transfected into macrophages (Supplementary Document 7). Of these 3’ UTR sequences, mutations in three miRNA binding sites significantly enhanced expression of the upstream reporter luciferase (*P*<0.05) indicating that the miRNAs could repress the expression of the reporter gene by pairing with the predicted target sites (Figure 4). In brief, both mutations in the *miR-221* binding site in the 3’ UTR of FOS induced expression of the upstream Renilla luciferase at 48 h post transfection. Both mutations in the *miR-27b* binding site in the 3’ UTR of the IFNG up-regulated the expression of the upstream Renilla luciferase at 48 h post transfection. Both mutations in the *miR-125b* binding site of the 3’ UTR of MAPK14 induced expression of the upstream Renilla luciferase at 48 h post transfection. Interestingly, one mutation in the *miR-221* binding site in the 3’ UTR of the FOS also significantly induced the expression of the upstream Renilla luciferase at 24 h of post transfection. One mutation in the *miR-125b* binding site in the 3’ UTR of MAPK14 significantly induced expression of the upstream Renilla luciferase at 24 h post transfection.

**Figure 5:**
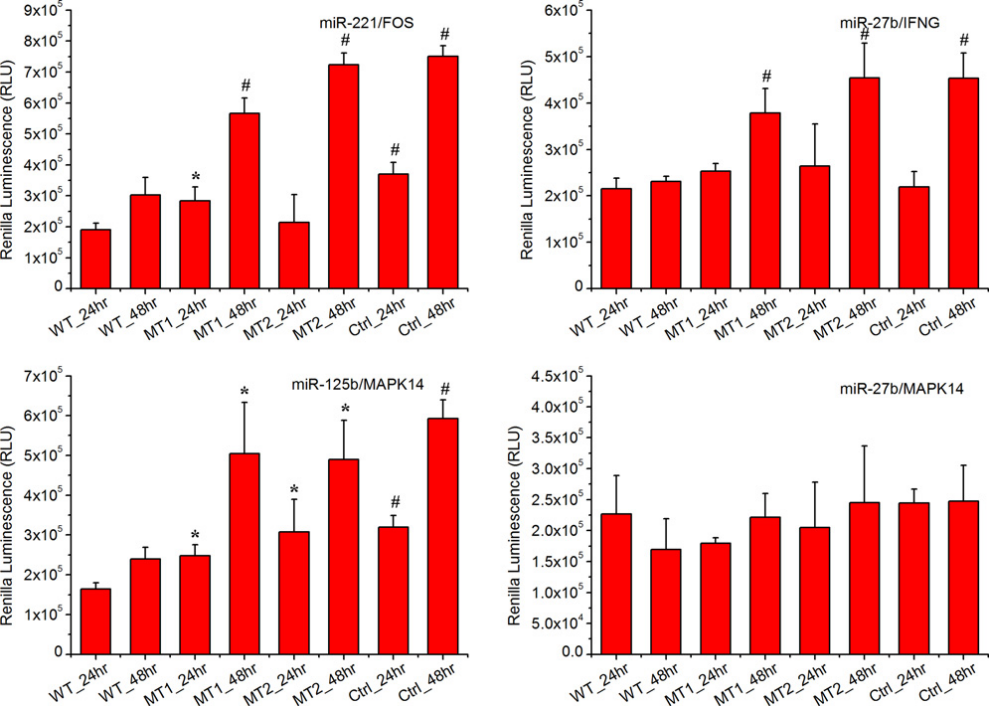
Experimental validation of the predicted targets in PBMCs One WT construct, two mutant (MT1 and MT2) constructs and one control (Ctrl) construct (does not had the 3’ UTR fragment of the target gene) were investigated. Statistically significant differences compared with the WT construct are visualized as *P≤0.05, ^#^*P*≤0.01.

However, it is problematic to use a mouse macrophage cell line (RAW 264.7) to validate the potential targets of selected miRNAs in pig. Thus, another experiment using peripheral blood mononuclear cells (PBMCs) from pig was performed to further validate the hypnosis Figure 5. Again, both mutations in the *miR-221* binding site in the 3’ UTR of FOS induced expression of the upstream Renilla luciferase at 48 h post transfection; both mutations in the *miR-27b* binding site in the 3’ UTR of the IFNG up-regulated the expression of the upstream Renilla luciferase at 48 h of post transfection. Both mutations in the *miR-125b* binding site of the 3’ UTR of MAPK14 induced expression of the upstream Renilla luciferase 48 h post transfection. Slight differences was observed in the Renilla luciferase signal at 24 h post transfection, and both mutations in the *miR-125b* binding site of the 3’ UTR of MAPK14 significantly induced expression of the upstream Renilla luciferase. The first mutation (MT1) for *miR-221* binding site in the 3’ UTR of the FOS significantly induced the expression of the upstream Renilla luciferase but the second mutation (MT2) does not. The luciferase reporter activity transfected with the WT 3’ UTR region was significantly lower than that in cells transfected with the control plasmid (without the 3’ UTR region), and the mutations of the binding site in the 3’ UTRs attenuate this effect.

In three of the four cases tested (*miR-221*/FOS, *miR-27b*/IFNG and *miR-125b*/MAPK14), the sites identified by TargetScan influenced expression of an upstream ORF when expressed in the same cells as the corresponding miRNAs. One remaining gene (*miR-27b*/MAPK14) is either not a valid target or has targets whose regulation was not detected in our assays. Regulation would be missed in cases for which the required cell type specific factors were not presented in macrophage cells or in cases when additional target site sequence environments were required but not included in the 3’ UTR segments used in our reporters. Thus, an alternative assay should be used to validate these hypotheses.

## DISCUSSION

In the present study, we tried to identify miRNAs that play a role in the Salmonella swine infection pathway. The miRNAs highly conserved across closely related species were used to predict pig miRNA candidates [34]. miRNA target genes in the Salmonella infection pathway were predicted using computational approaches and then validated via wet lab experiments. We identified miRNAs and target genes that mediate the effect of Salmonella infection using the following criteria: (1) the miRNA was predicted target genes involved in the Salmonella infection pathway; (2) the miRNA is expressed in either peripheral blood or lymph nodes; (3) the miRNA is differentially expressed in the Salmonella inoculation experiment in peripheral blood samples and (4) the miRNA regulation of its target gene can be validated by the luciferase reporter assay. Using these criteria, we found three miRNA/target interactions that are involved in Salmonella infection. We refer to these as ‘confirmed targets’.

Peripheral blood samples are a useful and practical window into the status of the immune system in health and disease [35,36]. Our previous study identified genes that mark early differential immune responses to Salmonella in pigs by correlating Salmonella infection and peripheral blood mRNA responses. Here, we further explored the miRNAs that play a role in the Salmonella infection pathway and investigated the expression of those miRNAs in peripheral blood. Analysis of peripheral blood would be very valuable as a low cost means for measuring miRNAs whose expression patterns are associated with a Salmonella infection. Thus, large numbers of individuals can be tested. The identified early miRNA biomarker associated with Salmonella infection could potentially be used to select animals that are less susceptible to infection, and thus serve as a useful means to improve pig genetics and food safety problems due to Salmonella infection.

*miR-125b* is the most attractive of the miRNA identified in this experiment that involved in the Salmonella infection pathway. It has been reported that the *miR-125b* plays a role in the innate immune response. LPS stimulation of mouse Raw 264.7 macrophages resulted in the down-regulation of *miR-125b* levels [37]. Tili et al. showed that *miR-125b* targets the 3’ UTR of TNF-α transcripts and therefore its down-regulation in response to LPS may be required for proper TNF-α production [37,38]. Using an integrative screening strategy, Kim et al. [39] found that the negative NF-κB regulator TNFAIP3 is a direct target of *miR-125b*, which suggested that the presence of a positive self-regulatory loop whereby suppression of TNFAIP3 function by *miR-125* could strengthen and prolong NF-κB activity.

Using northern blotting and real-time PCR technology, we found that *miR-125b* was significantly suppressed in the peripheral blood samples after Salmonella inoculation. However, only one gene in the Salmonella infection pathway, MAPK14, was predicted to target *miR-125b*. This target was further validated by luciferase reporter assay. MAPK14 is a MAP kinase and is an integration point for multiple biochemical signals that are involved in a wide variety of cellular processes. In the Salmonella infection pathway, MAPK14 is a downstream mediator of the Rho family GTPases CDC42. The stimulation of CDC42 leads to activation of the MAPK signalling pathways as well as transcription factors (through MAPK14) resulting in the reprogramming of host cell gene expression and the production of inflammatory cytokines [40–42]. Our results indicated that *miR-125b* is a direct regulator of the CDC42 effects, and significantly down-regulating *miR-125b* will release the inhibition of MAPK14 and enhance the downstream MAPK signalling pathway.

Two other miRNAs, *miR-221* and *miR-27b*, are also important in swine Salmonella infection. Using *in silico* target prediction analysis and experimental validation, Hu et al. [43] revealed the relationship of *miR-221* with the 3’ UTR region of ICAM1 mRNA and resulted in translational repression. They showed that IFN-γ stimulation decreased *miR-221* expression, and *miR-221*-mediated expression of ICAM1 showed a significant influence on the adherence of co-cultured T-cells. This suggests an important role for *miR-221* in the regulation of inflammatory responses. Buck et al. [44] reported a rapid down-regulation of *miR-27b* in multiple mouse cell lines as well as primary macrophages upon infection with the murine cytomegalovirus. They demonstrated that *miR-27b* exerts an antiviral function for over expression. Jennewein et al. showed that mutation of a *miR-27b* binding site within the 3’ UTR restored luciferase activity of PPARG. This indicates that *miR-27b* was involved in the inflammatory response [45].

Here, the *miR-221* was predicted to target one protein-coding gene—the FOS (AP-1). The *miR-27b* was predicted to target two protein-coding genes, IFNG and MAPK14, and both of these genes were significantly and differentially expressed in peripheral blood after Salmonella inoculation [13]. Interestingly, one of the targets of *miR-27b*, the MPAK14, is also predicted to target *miR-125b*, which was described above. Our luciferase reporter assay validated that FOS is a target of *miR-221* and IFNG is a target of *miR-27b*. The interaction between *miR-27b* and MAPK14 was not validated in our experiment.

In the Salmonella infection pathway, FOS is also a downstream mediator of the Rho family GTPases CDC42. The stimulation of CDC42 activates the extracellular signal-regulated kinases (ERKs) [42]. The activation of the FOS is one of the earliest nuclear events induced by growth factors, which stimulate ERKs [46,47]. Our results indicated that *miR-221* is a direct regulator of the FOS effects, although significantly down-regulation of *miR-221* will increase the effects of ERKs and enhance the activity of the MAPK signalling pathway. The activation of MAPK signalling pathway will promote the pro-inflammatory response, chemo attraction, proliferation, differentiation—and most importantly—the IFN-γ secretion [48]. In a previous study, we showed that IFN-γ is a major regulator of genes differentially expressed during the salmonella inoculation experiment [13]. In this experiment, the *miR-27b* was proven to regulate IFN-γ and its lower expression will produce IFN-γ.

## CONCLUSION

In the present study, miRNAs participating in the Salmonella infection pathway were identified via *in silico* prediction plus a homology search pipeline. They were then validated via an experimental approach. This work suggests that a number of miRNAs in the Salmonella infection pathway and *miR-221*, *miR-125b* and *miR-27b* played an important role. The findings regarding miRNAs and their peripheral blood-specific targets are helpful to understand the function of pig miRNAs in the future. Furthermore, the expression level of the miRNAs analysed across peripheral blood samples collected at pre- and post-natal stages of Salmonella inoculation in pig may be worthy of further investigation in terms of the biological events leading to an immune responses in pig.

## Abbreviations

ncRNA: noncoding RNA
TLR: toll-like receptor
RIN: RNA integrity number
WT: wild type
